# Effect of Peer Health Workers on AIDS Care in Rakai, Uganda: A Cluster-Randomized Trial

**DOI:** 10.1371/journal.pone.0010923

**Published:** 2010-06-02

**Authors:** Larry W. Chang, Joseph Kagaayi, Gertrude Nakigozi, Victor Ssempijja, Arnold H. Packer, David Serwadda, Thomas C. Quinn, Ronald H. Gray, Robert C. Bollinger, Steven J. Reynolds

**Affiliations:** 1 Division of Infectious Diseases, Johns Hopkins School of Medicine, Baltimore, Maryland, United States of America; 2 Rakai Health Sciences Program, Kalisizo, Uganda; 3 Johns Hopkins University, Baltimore, Maryland, United States of America; 4 Laboratory of Immunoregulation, Division of Intramural Research, National Institute for Allergy and Infectious Diseases, National Institutes of Health, Bethesda, Maryland, United States of America; 5 Johns Hopkins Bloomberg School of Public Health, Baltimore, Maryland, United States of America; Tulane University, United States of America

## Abstract

**Background:**

Human resource limitations are a challenge to the delivery of antiretroviral therapy (ART) in low-resource settings. We conducted a cluster randomized trial to assess the effect of community-based peer health workers (PHW) on AIDS care of adults in Rakai, Uganda.

**Methodology/Principal Findings:**

15 AIDS clinics were randomized 2∶1 to receive the PHW intervention (n = 10) or control (n = 5). PHW tasks included clinic and home-based provision of counseling, clinical, adherence to ART, and social support. Primary outcomes were adherence and cumulative risk of virologic failure (>400 copies/mL). Secondary outcomes were virologic failure at each 24 week time point up to 192 weeks of ART. Analysis was by intention to treat. From May 2006 to July 2008, 1336 patients were followed. 444 (33%) of these patients were already on ART at the start of the study. No significant differences were found in lack of adherence (<95% pill count adherence risk ratio [RR] 0.55, 95% confidence interval [CI] 0.23–1.35; <100% adherence RR 1.10, 95% CI 0.94–1.30), cumulative risk of virologic failure (RR 0.81, 95% CI 0.61–1.08) or in shorter-term virologic outcomes (24 week virologic failure RR 0.93, 95% CI 0.65–1.32; 48 week, RR 0.83, 95% CI 0.47–1.48; 72 week, RR 0.81, 95% CI 0.44–1.49). However, virologic failure rates ≥96 weeks into ART were significantly decreased in the intervention arm compared to the control arm (96 week failure RR 0.50, 95% CI 0.31–0.81; 120 week, RR 0.59, 95% CI 0.22–1.60; 144 week, RR 0.39, 95% CI 0.16–0.95; 168 week, RR 0.30, 95% CI 0.097–0.92; 192 week, RR 0.067, 95% CI 0.0065–0.71).

**Conclusions/Significance:**

A PHW intervention was associated with decreased virologic failure rates occurring 96 weeks and longer into ART, but did not affect cumulative risk of virologic failure, adherence measures, or shorter-term virologic outcomes. PHWs may be an effective intervention to sustain long-term ART in low-resource settings.

**Trial Registration:**

ClinicalTrials.gov NCT00675389

## Introduction

The provision of antiretroviral therapy (ART) in low-resource settings entails substantial challenges due to human resource limitations [1]. One of the main strategies advocated by the World Health Organization (WHO) and the United States President's Emergency Plan for AIDS Relief (PEPFAR) to address this crisis is through task shifting, the rational redistribution of tasks among health workforce teams from higher trained providers to those who require less training [2]. Community health workers (CHWs) are a key cadre to whom tasks can be shifted; however, there is limited trial-based evidence on their effectiveness in improving AIDS care outcomes [2], [3].

Community-based peer health workers (PHWs) are people living with HIV (PLHIV) and may potentially be a valuable type of CHW. Peers have been used effectively in HIV/AIDS programs in low-resource settings, typically as peer educators [4], and psychosocial support using peers has been recommended by the WHO for all PLHIV [5]. However, PHWs could deliver more care-oriented services in addition to counseling, education, and social support, and may therefore provide one strategy to mitigate the human resource crisis.

The Rakai Health Sciences Program (RHSP) is located in the rural Rakai District in southwest Uganda. Since June 2004, PEPFAR has enabled RHSP to provide ART via a decentralized, mobile clinic approach. In an operational and implementation research effort to evaluate the role of task shifting with PHWs at RHSP [6], [7], we conducted a cluster-randomised trial of the effect of PHWs on adult AIDS care outcomes [8]. Descriptive pilot data were previously presented [9], and this study reports trial outcomes. Our primary hypothesis was that, compared to patients in control communities, patients on ART in communities with PHWs will have improved adherence and fewer virologic failures.

## Methods

The protocol for this trial and supporting CONSORT checklist are available as supporting information; see Protocol S1 and Checklist S1.

### Ethics Statement

The trial was approved by institutional review boards at the Uganda Virus Research Institute, the Uganda National Council for Science and Technology, Johns Hopkins University, and the Western Institutional Review Board (Olympia, WA). Informed consent was not obtained for this study as the institutional review boards agreed that (i) PHWs were program staff performing routine care functions, and (ii) only de-identified programmatic data would be analyzed by community of randomization, and therefore no informed consent was required.

### Study Setting

The Rakai district in southwestern Uganda has a population of approximately 460,000 persons in an area of about 5000 square kilometers. In June 2004, RHSP/PEPFAR began providing ART through a mobile clinic program operating in 15 non-overlapping catchment areas (clusters) throughout the district. The mobile clinic model consisted of medical staff traveling from a central facility to designated government health clinics in each catchment area biweekly. In between clinic days (13 out of 14 days), patients' options for accessing care were limited and included traveling to a central facility, calling an RHSP mobile phone hotline (with call cost paid by caller) or toll-free warmline (similar to a hotline but staffed only during clinic hours), or visiting non-RHSP care facilities and providers [10].

### Participants

This trial was conducted between May 2006 to July 2008 and comprised all adult patients at the 15 mobile clinic sites who were either already on ART at the start of the trial or were started on ART at any time during the trial. About half (53%) of these patients were referred to the clinic from previous or current RHSP studies. All of these studies have recruited participants representative of Rakai District as a whole [11]. The remaining participants (47%) were “walk-ins” as any Rakai resident could come to these clinics and receive HIV counseling, testing, and care. Eligibility criteria for starting ART was a CD4 count ≤250 or WHO Stage IV illness [12]. All care and medications were provided free of charge.

### PHW Intervention (Arm A)

In addition to the usual standard of care at all clinic sites, Arm A clinics received the PHW intervention. The general approach to the design and implementation of this intervention was pragmatically-oriented, meaning that a general framework for PHW recruitment, training, tasks, and monitoring was developed, but the intervention was allowed to adapt to needs and problems which arose, e.g. arranging for a home visit to occur at a worksite if so requested by a patient [8]. Criteria for becoming a PHW included being a PLHIV on ART, good ART adherence for at least six months, and literacy. PHWs were nominated by fellow patients at each clinic site with final approval by RHSP staff if they met all qualifications. PHWs received a two day residential training on basic HIV pathogenesis, prevention, treatment, adherence counseling, performing pill counts, protecting patient confidentiality, and filling out a home visit form. Trainers included RHSP staff and experienced PHWs from an urban-based Ugandan ART program [13]. At the clinic, PHW tasks included providing ART counseling and support in group and individual sessions. For their home visit tasks, PHWs were initially assigned about 15 patients each who were visited biweekly. At these visits, PHWs were tasked to record on a standardized form a review of symptoms, a patient self-report of adherence, and to perform and record a pill count. PHWs were asked to counsel and educate their patients on ART adherence and general HIV/AIDS-related issues during these home visits. If patients were thought to need urgent care, PHWs were asked to alert RHSP staff and facilitate transfer to a higher level of care. PHWs returned completed forms to subsequent clinic sessions where they were added to patient charts for provider review. To assist with their duties and encourage retention, PHWs were each given a bicycle, identifying t-shirts, basic supplies, and an initial monthly allowance of about 12.5 USD. Day-to-day supervision of PHW activities were largely performed by a single RHSP staff member working part-time.

### Control Group (Arm B)

The control arm continued with the usual standard of care. However, standard of care did change over the study period, as a number of changes unrelated to the PHWs were subsequently implemented by RHSP in both the PHW and control arms. These programmatic changes included a peer educator program to promote use of preventive care services in mid-2006, the use of viral load results to guide care in late 2006, more focused ART-related health messaging in early 2007, and the use of enhanced adherence counseling, chart stickers to help identify patients failing virologically, and second-line ART provider talks in mid- to late 2007.

### Mobile Phone Support Intervention Substudy (Arm A^1^ and A^2^)

As a substudy, PHW intervention areas were also randomized 2∶3 to receive a mobile phone support intervention (Arm A^1^, n = 4 clusters) or not (Arm A^2^, n = 6 clusters). PHWs randomized to the mobile phone intervention were each given a mobile phone and, in addition to their usual responsibilities, were tasked to use text messaging to send home visit data back to the central clinic to be reviewed by centralized staff. PHWs could also call providers with questions or concerns [10]. Detailed results from this substudy will be presented elsewhere.

### Procedures

We used an unrestricted randomization process. The 15 mobile clinic sites were randomized 2∶1 to receive the PHW intervention (Arms A, n = 10 clusters) or control (Arm B, n = 5 clusters). We assigned clusters using unmatched, unrestricted random allocation by a drawing of lots. Study investigators (LWC, JK) generated the allocation sequence and implemented the randomization. This study was open label and unblinded.

### Outcomes

The primary outcomes included adherence (pill counts) and cumulative risk of virologic failure (any failure during follow-up period equaling failure). Secondary outcomes were virologic failure at each 24 week time point up to 192 weeks of antiretroviral therapy, mortality, lost to follow-up, and CD4 change at 24 and 48 weeks of ART. A summary clinic pill count was calculated by dividing the number of pills taken over the study period by the sum of pills expected to be taken over the study period and was analyzed dichotomously using two adherence cut points, 95% and 100% [14]. A patient self-report of adherence was also collected with patients reporting medication doses missed over the three preceding days at clinic visits. This self-report outcome was analyzed dichotomously, i.e. any self-report of missed doses. Viral loads (failure defined as >400 copies/mL) were measured at 24 to 192 week time points from antiretroviral therapy initiation by 24 week intervals. Progressively lower numbers of virologic outcomes available for analyses over time resulted from administrative censoring as patients started ART at different time points and only outcomes occurring during the study period were analyzed. As some patients began ART prior to the study period, these patients may have had early virologic outcomes prior to the trial which were not considered in analyses. Analyses were both stratified and combined by patients initiating ART prior to the trial and initiating during the trial. Viral loads were considered during the study period if they were collected one month after the start of the PHW intervention to help account for a “wash-out” period during which viral loads would have been unlikely to be reflective of intervention effects. Viral loads and CD4 counts were performed every 24 weeks on all patients as part of routine patient monitoring procedures. HIV viral loads were measured using the Amplicor Monitor Assay, version 1.5 (Roche Diagnostics, Branchburg, NJ, USA) with a lower limit of detection of <400 copies/mL. CD4 cell counts were measured using FACSCount or FACSCalibur (Becton Dickinson, San Jose, CA, USA). Patients were considered lost to follow-up if they had not had a pharmacy visit for medication pick-up in over 90 days. Mortality was ascertained through verbal autopsies.

### Statistical analysis

We originally estimated about 1000 patients would contribute outcomes to this study and made our power calculations assuming a 10∶5 ratio of intervention to control clusters with balanced numbers of participants in each cluster and no matching. Assuming a 5% drop out rate, we anticipated approximately 4,909 person-weeks of follow-up information per cluster. Based on previous RHSP studies, we used a 24 week virologic failure risk of 28% (i.e. a rate of about 0.0137 failures/person-week) and a coefficient of variation (*k*) of 0.11 and intraclass correlation coefficient (r) of 0.0024 [15], [16]. With a two-sided alpha = 0.05 and 1-B = 0.80, comparing Arms A to B, the study was estimated to have the power to detect a reduction in cumulative virologic failure with a rate ratio of ≤0.74.

Efficacy analyses for cumulative risk of virologic failure and for virologic outcomes at specific time points from ART initiation were by intention to treat using log-binomial regression with generalized estimating equations (GEE), an exchangeable correlation structure, and robust variance estimates appropriate for cluster-randomised trials [17]. To address potential concerns with multiple testing, we conducted a global test for an overall difference in relative risks across all time points between the two arms using a GEE model with a Wald test statistic. Additionally, we conducted trend analyses for differences in risks in the intervention and control arm over time using interaction terms. Finally, we conducted time to event analyses for first virologic failure using Cox proportional hazards modeling corrected for clustering. Because participants could have multiple episodes of failure and suppression and analyses such as time to event did not capture this clinical complexity, we focused on reporting the log-binomial analysis at each time point from ART initiation. This analysis allowed determination of risk ratios for all time points, fully utilized all available data, and better reflected the clinical and programmatic complexity of ART. Analyses were done with SAS 9.2 (SAS Institute Inc., Cary, NC) and STATA v10 (StataCorp, College Station, TX).

## Results

In April and May 2006, 19 PHWs were recruited, trained and deployed at the ten intervention clinics; one to three PHWs were assigned per clinic depending on patient load. Due to growing patient numbers, a second group of 10 PHWs were deployed in June 2007. Over the study period, the patient to PHW ratio grew from a mean of around 15∶1 (range 9–26∶1) to about 28∶1 (range 18–42∶1). Process evaluations found that PHWs had visited 96% of eligible patients at least once and <1% of patients were known to have refused PHW visits entirely. Based on completed home visit forms, PHWs made about 11,768 home visits over 26 months, averaging approximately 13 total visits per patient in the PHW arm at a rate of about 1.1 visits per patient per month. Each PHW visited approximately 5.4 patients per week.

**Figure 1:**
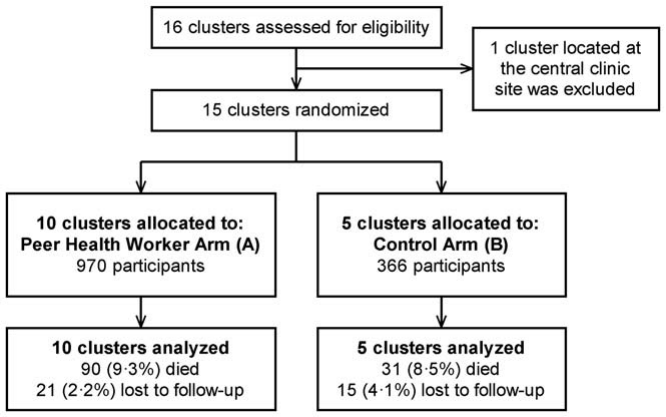
Trial profile.

**Figure 2:**
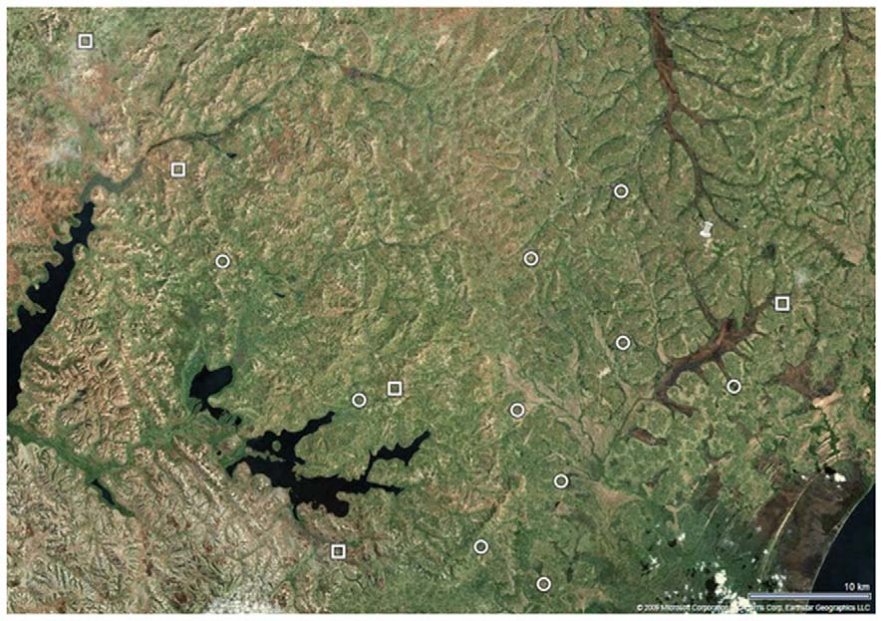
Map of cluster sites in Rakai, Uganda. Legend: Circles = Peer Health Worker Intervention Clinics (Arm A); Squares = Control Clinics (Arm B); Thumbtack = Central Clinic.

Figure 1 shows the flow of participants through the study. Figure 2 shows a map of the Rakai area and the clinic sites. At the start of patient follow-up in May 2006, 444 active patients were receiving ART through the RHSP/PEPFAR clinics (330 in Arm A, 114 in Arm B). By the end of the study period in July 2008, 892 additional patients had been started on ART (640 in Arm A, 252 in Arm B), giving a total of 1336 patients (970 in Arm A, 366 in Arm B) with some follow-up during the study period. Table 1 shows enrollment characteristics. Sociodemographic characteristics, immunologic and clinical stage of disease, the proportion of patients on ART, the median duration of time patients were on ART prior to the start of the trial, and the pre-trial 24 week and 48 week virologic failure rates appeared well balanced between arms.

**Table 1:** Characteristics according to randomization arm.*

Characteristic	Subcharacteristic	PHW Arm (A)	Control Arm (B)
No clusters		10	5
No subjects total		970	366
No subjects per cluster, mean (range)		97 (47–163)	73 (33–116)
Female, n (%)		638 (65.8)	247 (67.5)
Age, median (range), years		35.5 (15–76)	34.0 (17–70)
Age group, n (%)			
	≤29	244 (25.2)	102 (27.9)
	30–39	435 (44.9)	163 (44.5)
	≥40	291 (30.0)	101 (27.6)
CD4 cell count at ART initiation, median (IQR), cells/µl		160 (77–217)	161 (78–216)
CD4 groups at ART initiation, n (%), cells/µl			
	<100	292 (30.1)	101 (27.7)
	100–250	646 (66.6)	255 (69.9)
	>250	32 (3.3)	9 (2.5)
Plasma HIV-1 RNA at ART initiation, geometric mean, copies/mL		44440	36047
Plasma HIV-1 RNA at ART initiation, mean (SD), log_10_ copies/mL		4.65 (0.93)	4.56 (0.87)
Baseline viral load >100,000 copies/mL, n (%)		421 (56.1)	167 (61.4)
Baseline WHO Stage, n (%)			
	1	287 (29.6)	106 (29.0)
	2	349 (36.0)	140 (38.3)
	3	224 (23.1)	89 (24.3)
	4	109 (11.3)	31 (8.5)
Baseline ARV Regimen, n (%)			
	Combivir/Efavirenz	276 (28.5)	93 (25.5)
	Combivir/Nevirapine	353 (36.4)	156 (42.7)
	Stavudine/Lamivudine/Efavirenz	95 (9.8)	38 (10.4)
	Stavudine/Lamivudine/Nevirapine	239 (24.6)	74 (20.3)
	Other	7 (0.7)	4 (1.1)
Clinic distance to central clinic, mean (range), km		23.1 (7.7–40.5)	35.5 (8.6–54.5)
Subjects on ART prior to start of trial, n (%)		330 (34)	114 (31)
Subject pre-trial subject duration on ART, median (range), weeks		43.6 (1.0–89.4)	41.4 (0.6–89.6)
Pre-trial 24 week virologic failures, n/N (%)		85/191 (44.5%)	23/65 (35.4%)
Pre-trial 48 week virologic failures, n/N (%)		36/128 (28.1%)	12/46 (33.3%)

*PHW, Peer Health Worker; IQR, Interquartile Range; SD, Standard Deviation.

All 1336 patients had at least one adherence measure recorded, and 957 had at least one routine viral load result performed during the study period; 698/970 (72%) and 259/366 (71%) of patients in Arms A and B respectively had virologic outcome data. Median follow-up time for virologic outcomes in Arm A was 103 weeks per participant (interquartile range [IQR], 97-111 weeks) and in Arm B was 103 weeks (IQR, 94-113). Analyses of the primary outcome of cumulative risk of virologic failure showed that 17.8% of patients had at least one viral load failure in the PHW arm (124/698) compared to 21.6% in the control arm (56/259) which was nonsignificant (RR 0.81, 95% CI 0.61–1.08, p = 0.16).

Table 2 shows the secondary outcomes of rates of virologic failure during the study period by time on ART by 24 week intervals. Among all patients, the PHW intervention did not affect virologic outcomes ≤72 weeks into ART, but failure rates were generally lower in the intervention compared to the control arm after ≥96 weeks of ART. In stratified analyses by whether patients were initiated on ART before or after the start of the trial, no significant effect of the PHW intervention was observed among patients who initiated ART during the trial (all of whom had been on ART <120 weeks), but among those patients who initiated ART prior to the trial (81% of whom had a viral load at ≥120 weeks into ART), virologic failures were lower in the intervention compared to the control arm at most testing intervals ≥96 weeks.

**Table 2:** Estimates of effect for virologic outcomes.*

	PHW Arm (A)		Control Arm (B)		Arm A vs. B		
Week *x* of ART†	n failing/N	% failing	n failing/N	% failing	RR‡	95% CI	p value
***All subjects***							
**24**	45/462	9.7	18/173	10.4	0.93	0.65–1.32	0.68
**48**	42/456	9.2	18/164	11.0	0.83	0.47–1.48	0.54
**72**	21/384	5.5	9/138	6.5	0.81	0.44–1.49	0.59
**96**	26/398	6.5	17/134	12.7	0.50	0.31–0.81	0.005
**120**	18/272	6.6	10/87	11.5	0.59	0.22–1.60	0.30
**144**	12/212	5.7	10/68	14.7	0.39	0.16–0.95	0.039
**168**	6/131	4.6	6/39	15.4	0.30	0.097–0.92	0.035
**192**	1/85	1.2	5/27	18.5	0.067	0.0065–0.71	0.024
***Subjects initiating ART during trial***							
**24**	39/397	9.8	16/152	10.5	0.93	0.63–1.37	0.71
**48**	32/321	10.0	15/114	13.2	0.76	0.37–1.56	0.45
**72**	11/202	5.5	6/79	7.6	0.79	0.22–2.81	0.71
**96**	4/126	3.2	5/46	10.9	0.31	0.06–1.65	0.17
***Subjects initiating ART pre-trial***							
**24**	6/65	9.2	2/21	9.5	1.04	0.30–3.58	0.95
**48**	10/135	7.4	3/50	6.0	1.11	0.63–1.97	0.70
**72**	10/182	5.5	3/59	5.1	1.10	0.32–3.71	0.89
**96**	22/272	8.1	12/88	13.6	0.58	0.34–0.99	0.045
**120**	18/272	6.6	10/87	11.5	0.59	0.22–1.60	0.30
**144**	12/212	5.7	10/68	14.7	0.39	0.16–0.95	0.039
**168**	6/131	4.6	6/39	15.4	0.30	0.097–0.92	0.035
**192**	1/85	1.2	5/27	18.5	0.067	0.0065–0.71	0.024

*PHW, Peer Health Worker; RR, Risk Ratio; CI, Confidence Interval.

† Viral loads were done routinely on all patients every 24 weeks. Viral load results were included in these analyses only if they were performed during the study period.

‡ Risk Ratio calculated using generalized estimating equations with robust variances.

A global test for overall difference in relative risks across all time points was not significant (p = 0.16). Subgroup analysis of patients starting ART pre-trial showed a trend toward improved outcomes (p = 0.076) but analysis of those starting ART during the trial was not significant (p = 0.30). The trend analysis showed a statistically significant decline in the risk of virologic failures over time for the PHW arm compared to the control arm (p = 0.016). More specifically, in the PHW arm, the relative risk for virologic failure during the ≥96 week period compared to the ≤72 week period declined significantly (RR 0.44, 95% CI 0.24–0.78, p = 0.005), while in the control arm the relative risk trended in the opposite direction (RR 1.47, 95% CI 0.90–2.40, p = 0.12). Time to first virologic failure analyses did not find a significant decrease in time to first failure in the PHW arm (Hazard Ratio 0.82, 95% CI 0.56–1.21, p = 0.29). The intracluster correlation coefficient (ICC) for virologic failure at 24 weeks was 0.0015 based on analyses of 929 patients.

Table 3 shows estimates of effect for non-virologic outcomes, including the primary outcome of adherence. No significant differences were noted for these outcomes except for lost to follow-up rates which were improved in the PHW arm. Substudy analysis of the mobile phone support intervention among the PHWs found no statistically significant differences comparing Arm A^1^ to Arm A^2^ for all virologic and non-virologic outcomes (data not shown).

**Table 3:** Estimates of effect for non-virologic outcomes.*

Outcome	PHW Arm (A)		Control Arm (B)		Arm A vs. B	
	N	Outcome	N	Outcome	Estimate (95% CI)	p value
***All subjects***						
<95% pill count adherence, n (%)	874	12 (1.4)	330	8 (2.4)	0.55† (0.23–1.35)	0.20
<100% pill count adherence, n (%)	874	223 (25.5)	330	77 (23.3)	1.10† (0.94–1.30)	0.23
Any missed doses self-report vs. never, n (%)	898	158 (17.6)	338	65 (19.2)	0.99† (0.96–1.02)	0.60
Died, n (%)	966	90 (9.3)	366	31 (8**.**5)	0**.**99† (0**·**96–1**·**03)	0.60
Lost to follow-up, n (%)	966	21 (2.2)	366	15 (4**.**1)	0**.**56† (0.36–0.88)	0.01
***Subjects initiating ART during trial***						
CD4 Change at 24 weeks, mean (SD), cells/µl	415	155 (136)	156	157 (125)	−1.9‡ (−31.8−28.0)	0.90
CD4 Change at 48 weeks, mean (SD), cells/µl	331	189 (143)	116	197 (154)	−10.0‡ (−37.9−18.0)	0.49

*PHW, Peer Health Worker; CI, Confidence Interval; SD, Standard Deviation;

† Risk Ratios calculated from generalized estimating equations with robust variances;

‡ β_1_ from unadjusted general estimating equations model.

## Discussion

This trial found that a PHW intervention was associated with decreased virologic failure rates among patients on longer-term ART (≥96 weeks) with a significant trend of declining risk for virologic failures over time compared to the control arm. The PHW intervention was also associated with decreased lost to follow-up rates but had no effect on cumulative risk of virologic failure, virologic outcomes of patients on shorter-term ART, or adherence measures.

Counseling and support during the early ART initiation period was a major component of the PHW intervention, but these interventions did not have a significant effect on virologic outcomes of patients recently started on ART (≤72 weeks). Our study was originally powered to detect cumulative failure as a primary outcome rather than failure at individual time points, the rate of virologic failure was lower than anticipated, and virologic outcomes were not available for about 28% of participants, therefore this study was likely underpowered for these endpoints. Also, early ART users are likely to be highly motivated, and the PHW effect, if any, may be smaller during this period [18]. Additionally, the intensity of our intervention was less than we initially desired, i.e. higher patient to PHW ratios and less frequent home visits, which may have decreased the impact of the intervention. Insufficient power also likely affected our ability to detect differences with time to event analyses and global tests of effect.

However, point estimates for virologic outcomes favored the PHW Arm at all time points ≥96 weeks and generally trended downward over time. Only one outcome at these time points, at 120 weeks, was not statistically significant, likely due to a lack of power. This study did not find any differences in adherence, our second primary outcome, which may reflect a lack of sensitivity of our adherence measures. Lack of precision with self-report and pill-count adherence measures has been noted before [14]. Our number of outcomes for adherence <95% was also small, further complicating interpretation of these results. Loss to follow-up was significantly decreased in the PHW arm, consistent with other non-randomized trial studies which have noted the prominent role of CHWs in encouraging patient retention [19], [20].

The PHW association with improved virologic outcomes occurring after relatively longer periods of ART and the significant trend toward lower virologic failures over time suggest that PHWs may mitigate the effects of “treatment fatigue” (i.e. patients tiring of continually taking ART) [21]. Treatment fatigue may now represent a significant barrier to the optimal maintenance of effective ART in low-resource settings, and will likely grow in importance as experience with ART continues to accumulate. Notably, the PHW intervention effect was found only in patients who initiated ART pre-trial in stratified analysis, i.e. the group on ART longest, suggesting that this intervention may be best suited for patients who have taken ART for longer periods and are prone to treatment fatigue and its consequences.

Study limitations included its potentially limited generalisability as it was undertaken in the setting of an atypical, mobile clinic program nested in a long-standing research cohort. However, this model of decentralized care may be increasingly adopted in rural settings, and about half of patients in this ART program were “walk-ins” who had not previously participated in RHSP studies [22]. For this trial, process evaluations were performed as has been suggested is important to the understanding of complex interventions such as ours [23]. These evaluations found that while PHWs generally fulfilled their tasks, they did not visit patients as frequently as initially planned which may have blunted the intervention's effects.

We encountered a number of challenges and issues which may have relevance to the growing field of operations and implementation research in HIV/AIDS [6], [7], [24], [25]. For example, we disagree that there is no role for randomized trials in operations research [7], but rather, pragmatically-oriented randomized trials can be a useful study design to answer operations research questions. Also, our study design was notably influenced by programmatic resource constraints and priorities. For example, the randomization ratios were guided in part by programmatic concerns, and we were not able to increase the number of PHWs quickly enough to maintain our desired patient to PHW ratio. Additionally, other programmatic interventions implemented during the study period make interpretation of the effect of the PHW intervention more difficult. However, the significant associations of the PHW intervention with improved virologic outcomes in the setting of concurrent program improvements would tend to provide further support for a real intervention effect.

Furthermore, this study highlights the complexities of analyzing and understanding the effects of complex interventions implemented in the midst of an ongoing care program. For example, study patients initiated ART before and after the start of the intervention, and PHWs may have different impacts on these types of patients. Despite the challenges and limitations of this project, we believe operations and implementation research endeavors can result in important findings when pragmatically undertaken and cautiously interpreted.

PHWs represent a potentially sustainable work force that is unlikely to emigrate and able to remain proportional in size to the HIV epidemic [26]. PHWs are consistent with the WHO task shifting guidelines which encourage PLHIV to be part of the health workforce crisis solution [2], and the promotion of the greater involvement of PLHIV in their own care has been a longstanding policy of WHO and UNAIDS [27]. Further research on PHW processes, costs, training requirements, quality assurance, and supervisory needs are warranted. We previously reported early, rough costs of this intervention [9], and more rigorous cost analyses are planned.

In conclusion, a community-based PHW intervention was associated with decreased virologic failure rates occurring 96 weeks and longer into ART and decreased lost to follow-up rates but did not have an effect on cumulative risk of virologic failure, virologic outcomes of patients on shorter-term ART, or adherence measures. PHWs may offer a pragmatic and effective strategy for addressing the global human resource crisis in HIV/AIDS programs and promoting long-term sustainability of ART in low-resource settings.

## Supporting Information

Protocol S1 Trial Protocol.
(0.07 MB PDF)

Checklist S1 CONSORT Checklist.
(0.10 MB PDF)
