# Supplementary material for: Effect of Peer Health Workers on AIDS Care in Rakai, Uganda: A Cluster-Randomized Trial
Source: PLoS One. 2010 Jun 2;5(6):e10923. doi: 10.1371/journal.pone.0010923 (PMC2880005; doi:10.1371/journal.pone.0010923)
Supplement: Protocol S1 — Trial Protocol. (0.07 MB PDF) [file pone.0010923.s001.pdf]

Study Title: Impact of Peer Educators and Mobile Phones on HIV Care
Sponsor's Protocol # 2007014

Research Question:

Hypothesis

Study hypotheses include:

- Peer educators, by supporting adherence and by managing simple clinical issues, will reduce virologic treatment failure and improve ARV adherence compared to patients in communities without peer educators.
- Mobile phone technology used by peer educators, by more rapidly addressing adherence and clinical problems, will reduce treatment failure and improve adherence compared to patients in communities with peer educators without mobile phones.

Rationale:

Background

The provision of antiretroviral therapy (ART) in rural, resource-limited settings entails substantial challenges due to limitations in the health service infrastructure and in human resources for HIV/AIDS care.[1] In addition, long geographical distances between providers, care facilities, and patients can represent a significant barrier to appropriate and timely care. The use of peer educators as frontline adherence supporters in order to improve care in underserviced settings has been implemented by a number of programs, but the effect of peer support on HIV care outcomes has not been extensively evaluated.[2] Mobile phones have also been proposed as a potential method of improving access to health care in resource-limited environments by expediting communication and data transfer, but rigorous studies on their effectiveness in Africa have not yet been conducted.[3,4] Finally, Hotlines (staffed 24/7) and "Warmlines" (typically only staffed during clinic hours) have been used with success in the developed world to provide HIV/AIDS clinical advice to less experienced health care providers and patients; however, the these strategies have not been assessed in developing country settings.[6,7] Mobile phone coverage has expanded in Africa over the past 5 years,[5] providing an opportunity to improve communications between centrally located skilled health care providers and peripherally located staff, such as peer educators. Mobile phones offer the prospect of empowering peer educators by providing guidance and supervision to improve AIDS care in rural Africa.

The Rakai Health Science Project (RHSP) was founded in 1987 to study the HIV epidemic in the rural setting of Rakai District in southwest Uganda. Since June 2004, the US President's Plan for AIDS Relief (PEPFAR) has enabled the RHSP to provide ART through a community-based distribution system which includes clinical monitoring via a decentralized, mobile clinic approach. At this time, the program has screened 4,397 HIV-infected individuals and initiated ART in 849 patients. One of the challenges of providing ART in this setting has been the distance between many patients' homes and the clinic and medical staff trained in HIV care. This distance and the lack of communication channels make frequent clinic contacts difficult and has raised concerns about adherence, treatment failure and management of drug toxicity. Similarly challenges to ART delivery exist throughout rural Africa.[8] Adherence to ARV regimens is critical for prevention of treatment failure. [9-11]

The RHSP PEPFAR program has piloted the use of peer educators and mobile phone technology to optimize delivery of patient care and monitoring in this rural setting. The pilot peer educator program consists of nineteen persons living with HIV/AIDS (PLWHA) who are on ART, live in the community, and are literate. They are trained by the RHSP to visit patients in their homes biweekly or prn to help with issues of adherence and toxicity. Each peer educator supports 15-20 ART patients. Nine of the nineteen peer educators were provided with mobile phones and trained on using low-cost text messaging of patient clinical data and on calling a toll free RHSP Warmline.

Aim

This proposal builds upon the pilot peer educator, mobile phone, and Warmline project. In the first project year, we will conduct a three armed, community-randomized operations research trial to assess the effectiveness of peer educators, with and without mobile phones, in improving the delivery of HIV care in resource-limited settings. The three arms will be: a) communities with peer educators, b) communities with peer educators and mobile phones, and c) control communities without peer educators. The primary outcomes will be virologic treatment failure and drug adherence. Secondary outcomes will include change in CD4 count from baseline, morbidity (new opportunistic infection or drug toxicity), and mortality.

In the second project year we will scale up the peer educators and mobile phone technology to assess whether these interventions are sustainable, effective and appropriately cost effective. Qualitative studies will assess provider and patient attitudes towards these interventions.

Methods:

Study Design and Rationale

Research Design and Methods

Overview: A pilot peer educator/mobile phone intervention suggest that these programs are realistic in this resource-limited setting. However, there has been insufficient time and funding to assess the impact of these services on patient adherence and virologic outcomes. This proposal aims to build upon these early studies by:

1) Extending the cluster-randomized peer educator and mobile phone programs for a one year study period in order to better assess impact on virologic treatment failure and adherence. Secondary outcomes include CD4 count changes, mortality, and morbidity..

2) If a positive impact is demonstrated during the first project year, we will scale-up the peer educator and mobile phone interventions to all communities in order to evaluate the effects on compliance and virologic outcomes after introduction of services, compared to the prior time period. These interventions will be further evaluated by an analysis of cost and sustainability.

Three Arm Cluster-Randomized Operations Research Study:
Study Design and Population: The program will continue with the current three armed randomization scheme of 6 clinics with peer educators but no mobile phones (Arm A), 4 clinics with peer educators and mobile phones (Arm B), and 5 clinics with no peer

educators. We will maintain a ratio of 15-20 patients per peer educator. New peer educators will be recruited and trained as necessary to maintain this patient ratio. Outcomes will be compared between clinic patients in the peer educator arms compared to the control arm, as well as between patients in clinics with peer educators equipped with mobile phones compared to clinics with peer educators without mobile phones, or the control condition. All analyses will use de-identified data. The study population will be ~1000 PEPFAR patients enrolled in the RHSP ART program by March, 2007. Process data will be collected on intervention implementation. Provider evaluations will be done via PEPFAR staff and peer educator surveys.

Please note all patient records are routinely collected for clinical care so no additional consent is required and there is no risk to patients entailed by the evaluation, since de-identified data will be used.

Study Outcomes: The primary outcomes will be virologic failure (defined as viral load >400 copies/ml), and ARV adherence as measured by pill counts percentage (sum of ARV pills taken/sum of ARV pills prescribed). Secondary outcomes include change in CD4 count from baseline, morbidity (new opportunistic infection or drug toxicity), and mortality. These outcomes are recorded in clinical records and will be abstracted, and stripped of identifiers for analysis.

Data Collection: *Patient Data: As part of the clinical service, v*iral loads are assessed every six months and CD4 counts every three months for one year, and then every six months thereafter through PEPFAR clinical monitoring and a parallel NIH funded R01 study to evaluate the ARV effects on HIV epidemiology and behaviors (1R01HD050180; PI Maria Wawer). Data on ARV adherence will be derived from peer educator home visit records and pill counts at the bi-weekly clinic visits as part of the routine clinical service. Morbidity outcomes and descriptive data (gender, age, WHO stage, Karnofsky score) will be collected through clinic records and questionnaires administered by ARV clinic staff to patients initiating ART, weekly for the first month, and then at 3, 6, 9, 12, and 24 months on ART. These are routinely collected data and do not add a research burden. Mortality data will be collected through verbal autopsy interviews with relatives and friends of deceased patients. This is routinely done as part of the Rakai cohort study. Mobile phone data will be captured in real-time with an automated, preprogrammed SMS system. This mobile phone intervention will use locally available phones and technology.

Additional operational evaluation procedures for Peer Educators:
1) Every six months, a questionnaire with a Likert scale (ordinal scale graded from 1 to 5, e.g 1=Strongly Agree, 5=Strongly disagree) and free text (unstructured response) questions will be administered to the PEPFAR staff and peer educators on their impressions of the intervention and suggestions for improvement. 2) Peer educators will be required to demonstrate knowledge and skills with an oral and practical test following bimonthly, one-day continuing education sessions. 3) RHSP field staff will perform spot checks of peer educator home visits during the initial 2 months of implementation. 4) Peer educator home visit forms will be entered into an electronic database and descriptive statistics compiled. This is an ongoing evaluation of personnel for QA/QC purposes.

Population:

Sample Size

The total number of PEPFAR patients expected to be enrolled by May 2007 is ~1000, so we anticipate ~330 patients in each of the three study arms. We already have ~6 months follow up information on ~800 patients in the pilot study. With a further one year follow up we will have information over ~18 months. Assuming a 5% drop out rate, we anticipate ~5,643 person months (~470 py) per study arm. Detectable viral load (i.e., virologic failure) is ~28%.[12]

Power Calculations or Statistical Plan

This is an unmatched community randomized study. However, the Rakai communities have been selected to be homogeneous, and prior estimates indicate a coefficient of variation (k) between clusters of 0.24.[13] Thus, design effects are modest (~1.26). With a two-sided α = 0.05 and 1-β of 0.80, the study will have the power to detect a reduction in virologic failure in either intervention arm relative to the control arm with rate ratio (RR) ≤ 0.70 (i.e., a failure rate ≤ 19.6%). If the two peer educator intervention arms are compared to the control arm, the study will have a power to detect a reduction in virologic failure of RR ≤ 0.74 (i.e. ≤ 20.7%). Descriptive analyses will compare patients in each study arm at time of enrollment with respect to sociodemographic characteristics, WHO stage, viral load, CD4 count and Karnofsky score. Univariate analyses will assess outcomes by strata of covariates in each intervention arm. For dichotomous outcomes (e.g., virologic failure, drug toxicity, OIs, and mortality) we will use multivariate log-binomial regression to estimate the relative risk of treatment failure, adherence etc. between intervention arms, adjusted for covariates found to differ between arms at $p < 0.15$ in univariate analyses, or suspected confounders. Adjustment for clustered data will use general estimating equations (GGE) to estimate robust variances. For continuous data (e.g., adherence percent, CD4 changes from baseline to follow-up, number of drug toxicity events) we will use tests and analysis of variance (ANOVA). The effects of peer educators and mobile phone interventions will be assessed by stratified analyses and by interaction terms. The number of treatment failures prevented will be estimated.

Inclusion and Exclusion Criteria

Inclusion in the study is determined by receipt of ARVs in the PEPFAR program.
This protocol however deals only with the analysis of de-identified data and the intervention received by subjects takes place under a different service program.

Gender, Age and Locale

Both males and females between ages <1 and 60 are included.
The rural Rakai district in southwestern Uganda has an estimated population of ~460,000 persons in an area of over ~5000 square kilometers. In June 2004, with PEPFAR funding, antiretroviral therapy was provided through a community-based mobile clinic program operating in 15 localities. Each clinic has a catchment area of several villages, and the mobile clinic visits each location once every two weeks. Between visits to the mobile clinics, patients are supported by a “treatment companion” who is a friend or relative selected by the patient to assist in treatment support. The program has ~1000 active patients on ART with ongoing recruitment and a projected >1500 patients by the end of 2007. The ART program also has a central clinic staffed by physicians during the weekdays, but it is difficult for many patients to expeditiously reach this clinic when urgent issues arise.

Procedures:

Recruitment Process

Population will be ~1000 PEPFAR patients enrolled in the RHSP ART program by March, 2007.

Study Procedures in Sequential Order

See Mobile Phone Intervention Description below

Methods of Intervention

Peer Educator Intervention Description: Peer educators are themselves PLWHA on ART who have demonstrated good ART adherence for at least 6 months. Selection criteria include having a minimum of Primary 7 grade education and demonstrated literacy. Peer educators are nominated by their fellow patients at each clinic, approved by the field staff, and receive basic training on their roles and responsibilities. The peer educators are responsible for ~15-20 patients and are expected to visit the patients in their homes once every two weeks. At these visits, peer educators record a review of symptoms, client self-report of adherence, and a pill count. At the clinic, peer educators assist with patient organization and share their experiences, particularly with patients about to start ART. Peer educators undergo an initial, intensive two day residential training course and are provided with a bike and basic supplies, and a modest amount of remuneration to encourage compliance with their responsibilities and promote a high program retention rate.

Please note, Peer Educators are staff performing routine care functions, so no consent it required. Only de-identified data will be analyzed by community of randomization

Mobile Phone Intervention Description: The mobile phone intervention consists of the following: during home visits, peer educators with mobile phones, using data collected on their home visit forms, send a real-time text messages containing this clinical and adherence data back to the central clinic to be reviewed by clinical staff within a 24 hour period. This text message or SMS (Short Message Service) is a simple string of numbers derived from responses recorded on the coded home visit form. Peer educators may also call, toll-free, back to a central clinic Warmline with any questions or concerns. Clinicians at the central clinic receiving these text messages and phone calls may elect to provide care instructions to peer educators, send a higher level care provider to visit the patient, or arrange transport to the central clinic or hospital.

Methods for Dealing with Adverse Events

No AEs are anticipated as part of the Peer Educator project per se. The Peer Educators will notify medical personnel of any adverse events (e.g., drug toxicities, OIs), in order to provide care.

Methods for Dealing with Illegal Reportable Activities

There are no illegal reportable activities anticipated

Samples Stored Beyond the End of the Study

No samples will be collected as part of this study. All samples are collected under WIRB approved protocols for evaluation of the ART program.

Risk / Benefits:

Description of Risks

There are no risks with this protocol, only unidentified data will be available to the investigators.

Description of Measures to Minimize Risks

Peer Educators have been trained in maintenance of confidentiality. All patient identifiers (name, address, study ID#) will be stripped from the data set prior to analysis. Drs. Larry Chang and Ron Gray will be responsible for analysis and will only be provided with the anonymous data set stripped of all identifiers. They will not have access to any individual linkage information.

Description of Potential Benefits

No benefits to participants. Possible societal benefits if intervention improves outcomes.

Description of Level of Research Burden

No research burden. This is only analysis of de-identified program data.

Compensation:

Type of Compensation

There is no compensation for patients. The Peer Educators receive a small stipend and have access to a bicycle to support their work.

Amount of Compensation

N/A

Schedule of Compensation

N/A

Disclosure / Consent Process:

Description of the Consent Process

Waiver of consent

All HIV+ patients under care have provided consent for clinical services and have agreed to participate in a NICHD funded study of the Antiretroviral Program. No additional patient data is collected for this operations research study. The Peer Educators are staff who provide supportive services for patients, and this operations research study is an evaluation of the impact of their services so no consent is required.

Disclosure form will be provided.

Who Will Obtain Consent

Disclosure Form will be provided by Peer Educators

Where and When

At the time of home visit.

Confidentiality Assurances:

Certificate of Confidentiality (if applicable)

N/A

Data Security

The Rakai Project has, for 20 years, maintained full confidentiality of participant records. In keeping with Uganda Ministry of Health policy (please see below) the Rakai Program does not divulge individual HIV or other results to any third party, without the express written permission of the participant. In order to preserve confidentiality, informed consent documents are retained in locked filing cabinets and store rooms, accessible only to senior investigators or designated staff. All computerized data bases only contain study ID numbers. The lists linking study numbers to names is kept separately in a password protected computer, accessible only to senior data managers. Files of lab results are maintained in a separate safe computer file, with study ID numbers, and contain no personal identifiers.

Plans for Record Keeping

Records will be kept in secure filing cabinets and store rooms as described above.

Person Responsible & Telephone Number

Frederick Makumbi 256-0772 318387

Where Data Will Be Stored for Data Security

Kalisizo Field Station, Rakai district Uganda – locked store rooms.

Who Will Have Access to the Data

Data entry personnel, senior data managers and senior investigators.

Plans for Disposition of Identifiers at End of Study (state if identifiers will be stored or destroyed and the method that will be used to destroy the identifiers)

Identifiers will be maintained as long as patients are under care. If patients leave care (e.g., by death or drop out), their records will be retained for a minimum of 5 years after closure of the ART evaluation in 2012.

Plans for Destruction of the Data/Samples at the End of the Study (state the method that will be used to destroy the data (e.g. study documents, tapes, samples)

At this time, there are no plans for destruction of records since these are needed for patient care.

<u>Collaborative Agreements</u>:

Description of the Collaboration

Rakai Health Sciences Program (RHSP) has been collaborating with Johns Hopkins School of Public Health and PI Ron Gray for nearly 20 years. RHSP will be responsible for day to day operations of this study

Letter(s) of Collaboration

Attached

Name of Institution/Person

Uganda Virus Research Institute/ Rakai Health Sciences Program; PI: David Serwadda

Role of the Collaborative Organization

As above

Role of SPH Investigator

Oversight of activities and scientific design and guidance

<u>Other IRB Approvals:</u>

Uganda Science and Ethics Committee
